# Right ventricular dysfunction in patients with COVID‐19 pneumonitis whose lungs are mechanically ventilated: a multicentre prospective cohort study

**DOI:** 10.1111/anae.15745

**Published:** 2022-05-24

**Authors:** P. J. McCall, J. M. Willder, B. L. Stanley, C‐M. Messow, J. Allan, L. Gemmell, A. Puxty, D. Strachan, C. Berry, B.G. Shelley, COVID‐RV co‐investigators, A. Reece, C. Kitchen, M. Gillies, V. Dabek, V. Irvine, J. MacBrayne, K. Sim, T. Scott, E. Trumper, F. Savage, A. Allan, J. Falconer, A. Coutts, A. McDonald, J. Rutherford, D. Christie, C. Jardine, M. Hughes, S. Cathcart, M. Sim, B. Docking, M. Thornton, B. Greatorex, J. Rae, C. Barr, C. Bradley, F. Barrett, R. Campbell, N. Clarke, M. Mascarenhas, J. Matheson, D. McDonald, M. O'Hara, L. O'keeffe, R. Price, M. McHendry, D. McLaughlan, C. Herman, H. Elliot, S. Meehan, D. Finn, G. Brannan, S. Wood, T. Watson, K. Ross, N. Tatarkowska, R. Boyle, E. Lee, A. Morrison, Pl. Lucie, C. Lochrin, S. Clements, D. Vigni

**Affiliations:** ^1^ The Anaesthesia, Critical Care and Peri‐operative Medicine Research Group University of Glasgow UK; ^2^ Department of Anaesthesia Golden Jubilee National Hospital Clydebank UK; ^3^ West of Scotland School of Anaesthesia NHS Education for Scotland Glasgow UK; ^4^ Robertson Centre for Biostatistics University of Glasgow UK; ^5^ Robertson Centre for Biostatistics University of Glasgow UK; ^6^ Department of Intensive Care Medicine University Hospital Crosshouse Kilmarnock UK; ^7^ Department of Intensive Care Medicine Royal Alexandra Hospital Paisley UK; ^8^ Department of Intensive Care Medicine Glasgow Royal Infirmary Glasgow UK; ^9^ Department of Intensive Care Medicine University Hospital Wishaw UK; ^10^ Department of Cardiology and Imaging Institute of Cardiovascular and Medical Sciences, University of Glasgow UK; ^11^ The Anaesthesia, Critical Care and Peri‐operative Medicine Research Group University of Glasgow UK

**Keywords:** ARDS, COVID‐19, echocardiography, right ventricle

## Abstract

Cardiovascular complications due to COVID‐19, such as right ventricular dysfunction, are common. The combination of acute respiratory distress syndrome, invasive mechanical ventilation, thromboembolic disease and direct myocardial injury creates conditions where right ventricular dysfunction is likely to occur. We undertook a prospective, multicentre cohort study in 10 Scottish intensive care units of patients with COVID‐19 pneumonitis whose lungs were mechanically ventilated. Right ventricular dysfunction was defined as the presence of severe right ventricular dilation and interventricular septal flattening. To explore the role of myocardial injury, high‐sensitivity troponin and N‐terminal pro B‐type natriuretic peptide plasma levels were measured in all patients. We recruited 121 patients and 118 (98%) underwent imaging. It was possible to determine the primary outcome in 112 (91%). Severe right ventricular dilation was present in 31 (28%), with interventricular septal flattening present in nine (8%). Right ventricular dysfunction (the combination of these two parameters) was present in seven (6%, 95%CI 3–13%). Thirty‐day mortality was 86% in those with right ventricular dysfunction as compared with 45% in those without (p = 0.051). Patients with right ventricular dysfunction were more likely to have: pulmonary thromboembolism (p < 0.001); higher plateau airway pressure (p = 0.048); lower dynamic compliance (p = 0.031); higher plasma N‐terminal pro B‐type natriuretic peptide levels (p = 0.006); and raised plasma troponin levels (p = 0.048). Our results demonstrate a prevalence of right ventricular dysfunction of 6%, which was associated with increased mortality (86%). Associations were also observed between right ventricular dysfunction and aetiological domains of: acute respiratory distress syndrome; ventilation; thromboembolic disease; and direct myocardial injury, implying a complex multifactorial pathophysiology.

## Introduction

COVID‐19 can result in acute hypoxemic respiratory failure, which in severe cases may require admission to ICU and invasive mechanical ventilation. Haemodynamic instability and cardiac complications are prevalent, with right ventricular dysfunction a common finding [1, 2, 3, 4, 5, 6, 7]. The combination of acute respiratory distress syndrome (ARDS), invasive mechanical ventilation, thromboembolic disease and the potential for direct myocardial injury create an environment where right ventricular dysfunction is likely to occur [8]. In reports before the COVID‐19 pandemic, right ventricular dysfunction was thought to occur in up to 55% of patients with ARDS and had been found to be independently associated with mortality, with more severe ARDS associated with an increased frequency of right ventricular dysfunction [9, 10].

Previous echocardiographic studies in patients with COVID‐19 have demonstrated right ventricular dysfunction and highlighted its association with adverse clinical outcomes [2, 3, 4, 11]. However, much of the research into right ventricular dysfunction in COVID‐19 has been retrospective and performed in undifferentiated cohorts of patients whose lungs are or are not mechanically ventilated [4, 5, 6]. There have been no robust, prospective, haemodynamic evaluations of patients with COVID‐19 focusing specifically on the ICU population [3]. This study was designed as a pragmatic, critical care clinician‐led, prospective echocardiography study to elucidate the prevalence of right ventricular dysfunction in patients with COVID‐19 whose lungs are ventilated, its association with mortality and plausible causative mechanisms.

## Methods

The study protocol and methods have been reported previously [12]. We conducted a prospective, observational cohort study involving 10 ICUs in NHS Scotland. Ethics approval was obtained, as was informed consent from a legal representative for all patients. Patients were eligible for inclusion if they were aged > 16 y with confirmed SARS‐CoV‐2 infection, with severe acute respiratory failure requiring tracheal intubation and positive pressure ventilation in ICU. Study imaging was performed > 48 h after tracheal intubation and before day 14 of ICU admission. We did not study patients with any of the following criteria: pregnancy; ongoing participation in investigational research that may undermine the scientific basis of the study; previous participation in the study; requirement for extracorporeal membrane oxygenation; and end‐of‐life care, where the patient was not expected to survive > 24 h.

Study data were collected and managed using REDCap (Vanderbilt University, Nashville, TN, USA) electronic data‐capture tools hosted by the University of Glasgow. Baseline characteristics, chronic comorbidities, clinical trajectory before ICU admission, severity of illness, acute comorbidities and follow‐up data were all collected prospectively. Clinical data relating to potential mechanisms of right ventricular dysfunction were also collected, specifically regarding the four domains of: ARDS; disordered coagulation; myocardial injury; and mechanical ventilation.

Participants underwent a single transthoracic echocardiogram to determine the presence or absence of right ventricular dysfunction between day 2 and day 14 following tracheal intubation, with all other echocardiographic imaging performed at the discretion of treating clinical teams. To reflect the clinical practice of bed‐side echocardiography in ICU, and for the purposes of determining the primary outcome of the study, imaging was in keeping with the protocol for a focused intensive care echocardiography (FICE) scan [13]. The presence or absence of severe right ventricular dilation along with the presence or absence of interventricular septal flattening (in systole, diastole or both) was established. Severe right ventricular dilation was determined from the apical four‐chamber view at end‐diastole and was present when right:left ventricular area ratio > 1. High‐sensitivity troponin (I or T depending on assay used clinically at each site) and N‐terminal pro B‐type natriuretic peptide (NT‐proBNP) were measured in all patients on the day of echocardiography. Samples were processed alongside routine clinical samples in each host site and therefore subject to routine laboratory quality assurance processes. Raised values were defined for NT‐proBNP (> 300 ng.ml^‐1^) and troponin (TnT ≥ 15 ng.l^‐1^ or TnI ≥ 34 ng.l^‐1^ for males and ≥ 16 ng.l^‐1^ for females).

The primary outcome was the prevalence of right ventricular dysfunction and its association with 30‐day mortality [12]. Right ventricular dysfunction was defined as the presence of severe right ventricular dilation and interventricular septal flattening. Exploratory outcomes sought to determine associations between right ventricular dysfunction and proposed aetiological factors. In addition, associations between cardiac biomarker levels and 30‐day mortality were assessed. Sample size selection was, by necessity, a pragmatic balance of maximising available information vs. the delivery of the study. Given the number of patients admitted to participating ICUs in Scotland during the first wave of the pandemic (March–June 2020), we believed it was realistic to recruit 120–150 patients across participating sites. Power calculations were performed for estimated right ventricular dysfunction prevalence rates of 25% and 50%, with an overall mortality rate of 50% (see online Supporting Information Table S1). These analyses suggest that a study of 120 patients would have 80% power to detect an associated OR for mortality of 2.83–3.43 (with an estimated prevalence of right ventricular dysfunction of 50% and 25% respectively).

The proportion of patients with COVID‐19 whose lungs were ventilated with right ventricular dysfunction was determined, with a 95%CI utilising the Clopper–Pearson method. We then sought to analyse the association of right ventricular dysfunction with 30‐day mortality using logistic regression analysis predicting 30‐day mortality from presence or absence of right ventricular dysfunction, adjusting for baseline characteristics (age; sex; ethnicity), phase of disease (time from tracheal intubation to echocardiography) and baseline severity of illness (APACHE‐2 score). Firth's bias‐reduced logistic regression was used where ordinary logistic regression failed due to small numbers [14]. Ordinal and categorical data are presented as number (proportion). Between‐group differences were assessed using Fisher's exact test for categorical variables and Student's t‐test or Wilcoxon Mann–Whitney test for continuous variables. Statistical analyses were performed using R version 4.0.0 (R Foundation for Statistical Computing, Vienna, Austria).

## Results

**Figure 1:**
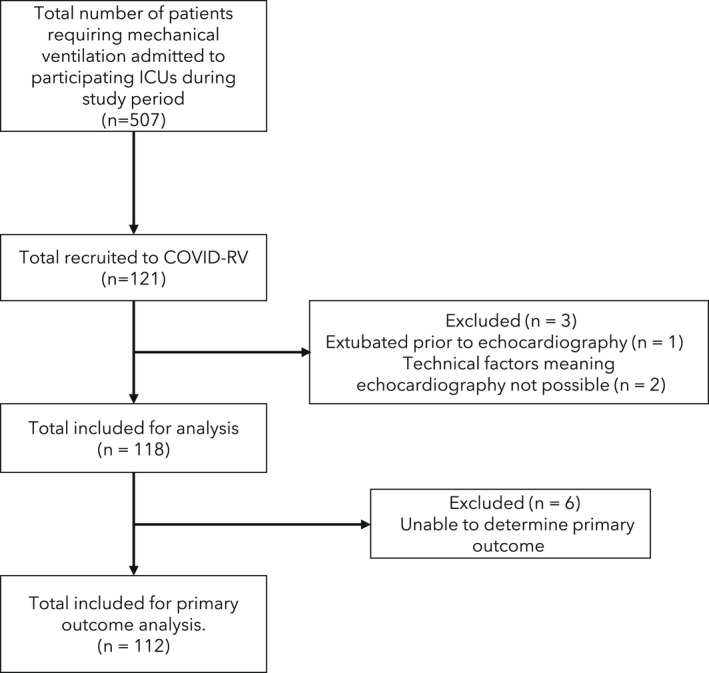
Patient recruitment and flow diagram.

Between 2 September 2020 and 22 March 2021, we recruited 121 patients (Fig. 1, Table 1). Following recruitment, three patients were excluded from further participation: one due to tracheal extubation before echocardiography and two due to technical factors preventing imaging. In the whole cohort, 30‐day mortality was 47%. Out of 118 patients where echocardiography was performed, it was possible to determine the primary outcome in 112 (95%). Echocardiography was performed by British Society of Echocardiography‐accredited echocardiographers in 55 (47%), FICE‐accredited critical care clinicians with ‘mentor’ status in 37 (31%), FICE‐accredited clinicians in eight (7%) and clinicians without any formal accreditation in 18 (15%). Imaging was performed a median (IQR [range]) of 5 (4–8 [2–14]) days following tracheal intubation.

**Table 1:** Characteristics of patients with COVID‐19 at the time of ICU admission. Values are mean (SD), number (proportion) or median (IQR [range]).

	All patients n = 112	No or less than severe right ventricular dysfunction n = 105	Severe right ventricular dilation and septal flattening n = 7	p value
Age; y	59 (11.3)	59 (11.6)	61 (4.4)	0.318
Sex; male	74 (66%)	69 (66%)	5 (71%)	> 0.99
BMI; kg.m^‐2^	33 (7.1)	33 (7.2)	32 (4.6)	0.467
Missing	2	2	‐	
Ethnicity				
White	100 (89%)	93 (89%)	7 (100%)	> 0.99
Non‐white	12 (11%)	1 (1%)	‐	
Time from symptom onset to tracheal intubation; days	11 (7–16 [0–46])	11 (7–14 [0–46])	18 (13–25 [7–33])	0.035
Clinical Frailty Score	2 (2–3 [1–5])	2 (2–3 [1–5])	2 (1.5–2.5 [1–3])	0.281
Missing	1	1	‐	
APACHE‐2 score	17(5.8)	17 (5.8)	18 (6.1)	0.687
Missing	5	5	‐	
Coronavirus clinical characterisation consortium mortality score	10 (2.7)	10 (2.8)	11 (2.3)	0.712
Missing	10	10	‐	
Smoking				
Non‐smoker	63 (56%)	61 (58%)	2 (29%)	0.228
Ex‐smoker > 1 year	40 (36%)	36 (34%)	4 (57%)	
Current or within 1 year	9 (8%)	8 (8%)	1 (14%)	
Alcohol				
None	34 (31%)	33 (32%)	1 (14%)	0.478
Minimal	57 (52%)	51 (50%)	6 (86%)	
Moderate	8 (7%)	8 (8%)	‐	
Excess	11 (10%)	11 (11%)	‐	
Hypertension	38 (34%)	36 (34%)	2 (29%)	> 0.99
Coronary artery disease	11 (10%)	11 (11%)	‐	> 0.99
Diabetes mellitus	33 (30%)	31 (30%)	2 (29%)	> 0.99
Asthma	16 (14%)	15 (14%)	1 (14%)	> 0.99
Chronic obstructive pulmonary disease	10 (9%)	10 (10%)	‐	> 0.99
Treatments before tracheal intubation				
Intravenous corticosteroids	74 (66%)	70 (67%)	4 (57%)	0.687
Non‐invasive positive pressure ventilation	76 (68%)	70 (67%)	6 (86%)	0.426
High‐flow nasal oxygen	65 (58%)	59 (56%)	6 (86%)	0.235
Awake self‐proning	57 (51%)	51 (49%)	6 (86%)	0.114
New arrhythmias	17 (15%)	14 (13%)	3 (43%)	0.07
Pulmonary thromboembolic disease				
Radiologically confirmed	4 (4%)	1 (1%)	3 (43%)	
Clinically suspected	5 (5%)	4 (4%)	1 (14%)	< 0.001
No	101 (90%)	98 (93%)	3 (43%)	
Unknown	2 (2%)	2 (2%)	‐	
Acute coronary syndrome	5 (5%)	5 (5%)	‐	> 0.99
Requirement for renal replacement therapy	18 (16%)	16 (15%)	2 (29%)	0.313

**Figure 2:**
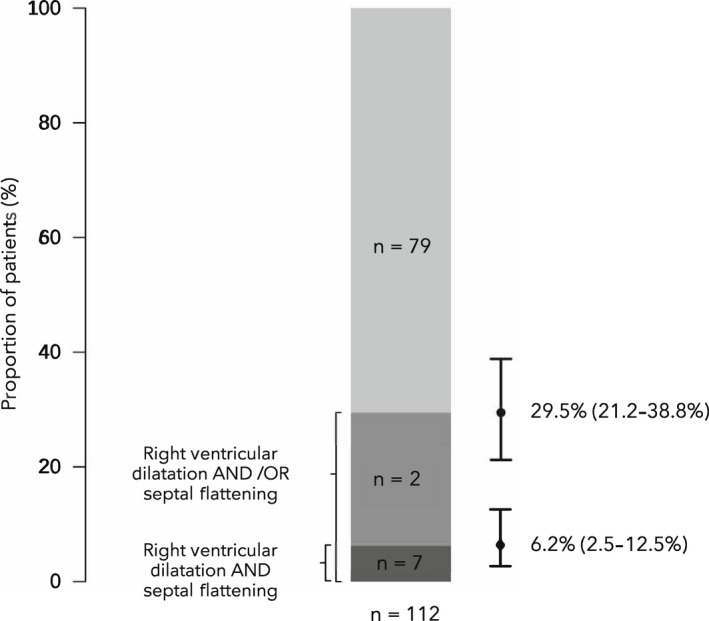
Proportions of patients with COVID‐19 whose lungs were mechanically ventilated and who had right ventricular dysfunction.

Thirty‐one (28%) patients had evidence of severe right ventricular dilation (right:left ventricular area ratio > 1:1) and nine (8%) had evidence of interventricular septal flattening. Right ventricular dysfunction (the combination of these two parameters) was present in seven patients (6%, 95%CI 3–13%) (Fig. 2). Subjective right ventricular dysfunction (determined qualitatively by the clinician performing echocardiography) was present in 86% with right ventricular dysfunction, in contrast to 10% of those without (p < 0.001). Subjective left ventricular dysfunction was present in 29% with right ventricular dysfunction, in contrast to 10% of those without (p = 0.168) (Table 2). There was no difference in time from tracheal intubation to imaging in patients with or without right ventricular dysfunction (p = 0.942). However, median (IQR [range]) time from symptom onset to echocardiography was longer in patients with right ventricular dysfunction as compared with without (23 (20–30 [15–37]) vs. 17 (13–21 [3–51]) days, p = 0.017, respectively (Table 2)).

**Table 2:** Measured echocardiographic variables of patients with COVID‐19 whose lungs were mechanically ventilated. Values are median (IQR [range]) or number (proportion).

	All n = 112	No or less than severe right ventricular dysfunction n = 105	Severe right ventricular dilation and septal flattening n = 7	p value
Time from symptom onset to echocardiography; days	18 (13–22 [3–51])	17 (13–21 [3–51])	23 (20–30 [15–37]	0.017
Missing	1	1	‐	
Time from tracheal intubation to echocardiography; days	5 (4–8 [2–14])	5 (4–8 [2–14])	5 (5–6 [2–11])	0.942
Right ventricular dilation	31 (28%)	24 (23%)	7 (100%)	< 0.001
Missing	2	2	0	
Septal flattening	9 (8%)	2 (2%)	7 (100%)	< 0.001
Missing	3	3	‐	
Subjective left ventricular dysfunction	12 (11%)	10 (10%)	2 (29%)	0.168
Missing	2	2	‐	
Subjective right ventricular dysfunction	16 (14%)	10 (10%)	6 (86%)	< 0.001
Missing	1	1	‐	

Patients with right ventricular dysfunction had a higher plasma creatinine at the time of imaging and were more likely to be undergoing renal replacement therapy (Table 3). Heart rate was higher in patients with right ventricular dysfunction and acid–base status was also more disordered, with lower plasma pH, lower base excess and lower bicarbonate (Table 3). Six (86%) patients with right ventricular dysfunction were deceased by the time of 30‐day follow‐up compared with 47 (45%) without (p = 0.051) (Table 4). When testing for independent association, Firth's bias‐reduced regression analyses controlling for baseline characteristics, phase of disease and baseline severity of illness showed the presence of right ventricular dysfunction was associated with an increased 30‐day mortality (OR 5.12, 95%CI 0.99–51.38; p = 0.051) (Table 5).

**Table 3:** Characteristics of patients with COVID‐19 whose lungs were mechanically ventilated on the day of echocardiography. Values are number (proportion), mean (SD) or median (IQR [range]).

	All n = 112	No or less than severe right ventricular dysfunction n = 105	Severe right ventricular dilation and septal flattening n = 7	p value
Mechanical ventilation in the prone position	79 (71%)	72 (69%)	7 (100%)	0.24
Sequential organ failure assessment score	7.9 (3)	7.8 (2.9)	9.3 (4.2)	0.408
Requirement for RRT on day of echocardiogram	15 (13%)	12 (11%)	3 (43%)	0.049
Arterial [H^+^]; nmol.l^‐1^	39 (36–46 [28–68])	39 (35–45 [28–63])	53 (53–61 [49–68])	0.001
Missing	15	13	2	
Arterial PaO_2_; kPa	9.3 (1.2)	9.3 (1.3)	8.8 (1.0)	0.219
PaCO_2_; kPa	7 (6–8 [4–13])	7 (6–8 [4–1])	8 (6–9 [5–13])	0.39
Missing	3	3	‐	
Arterial base excess; mmol.l^‐1^	5.9 (6.5)	6.4 (6.4)	‐1.2 (3.2)	< 0.001
Missing	2	2	‐	
Arterial bicarbonate; mmol.l^‐1^	32 (6.6)	32 (6.6)	26 (2.7)	< 0.001
Missing	3	3	‐	
Plasma haemoglobin; g.dl^‐1^	11 (1.8)	11 (1.8)	10 (1.2)	0.073
Plasma neutrophils; ×10^9^ l^‐1^	11 (9–15 [3–43])	11 (9–15 [3–43])	10 (8–13 [6–19])	0.512
Plasma lymphocytes; ×10^9^ l^‐1^	1 (1–1 [0–5])	1 (1–1 [0–5])	1 (1–2 [0–2])	0.341
Plasma platelets; ×10^9^ l^‐1^	280 (110)	282 (107)	243 (148)	0.518
Plasma C‐reactive protein; mg.l^‐1^	62 (12–158 [1–665])	60 (10–156) [1–665])	71 (29–186 [11–279])	0.528
Plasma D‐dimer; mg.l^‐1^	1264 (601–2605 [1–30,667])	1315 (573–2652 [1–30,667])	1156 (1105–1485 [890–2144])	0.961
Missing	31	29	2	
Prothrombin time; s	12 (11–13 [10–26])	12 [11–13 [10–26])	13 [11–15 [11–17])	0.434
Activated partial thromboplastin time; s	27 (25–31 [19–263])	27 (25–30 [19–263])	31 (27.1–36 [24–50])	0.12
Missing	1	1	0	
Plasma creatinine; μmol.l^‐1^	70 (54–107 [27–396])	67 (52–104 [27–396])	157 (112–212 [44–244])	0.014
N‐terminal pro B‐type natriuretic peptide; ng.l^‐1^	458 (199–1690 [36–61,280])	429 (197–1369 [36–31,136])	4806 (2571.5–17,121 [131–61,280])	0.006
Missing	12	12	0	
N‐terminal pro B‐type natriuretic peptide > 300 ng.l^‐1^	63 (63%)	57 (61%)	6 (86%)	0.255
Missing	12	12	0	
High‐sensitivity troponin I; ng.l^‐1^	12 (5–42 [0–3585])	11 (4–37 [0–3585])	56 (38–102 [6–278])	0.082
Missing	48	46	2	
High‐sensitivity troponin l; ng.l^‐1^	17 (10–29 [0–473])	16 (10–26 [0–473])	39 [34–44 [29–49])	0.094
Missing	66	61	5	
Raised plasma troponin	51 (46%)	45 (44%)	6 (86%)	0.048
Missing	2	2	0	
Heart rate; beats.min^‐1^	79 (19.9)	77.7 (19.5)	99.1 (17.3)	0.016
Rhythm				
Sinus	107 (96%)	100 (95%)	7 (100%)	> 0.99
Atrial tachycardia	5 (5%)	5 (5%)	0	
Mean arterial pressure; mmHg	81 (13.4)	81 (13.5)	74 (9.6)	0.1
Missing	3	3	0	
Central venous pressure; cmH_2_O	7 (4–12 [0–25])	7 (4–12 [0–20.4])	14 (10–17 [2–25])	0.187
Missing	38	35	3	
Vasopressors	40 (36%)	36 (34%)	4 (57%)	0.246
Inotropes	1 (1%)	1 (1%)	0	> 0.99
Anticoagulation				
Prophylactic	93 (83%)	90 (86%)	3 (43%)	0.018
Therapeutic	17 (15%)	13 (12%)	4 (57%)	
None	2 (2%)	0	0	
Neuromuscular blockade	52 (46%)	49 (47%)	3 (43%)	> 0.99
F_I_O_2_	0.6 (0.4–0.7 [0.3–1.0])	0.6 (0.4–0.7 [0.3–1.0])	0.6 (0.5–0.7 [0.4–0.8])	0.659
Requirement for prone ventilation in previous 24 h	44 (39%)	40 (38%)	4 (57%)	0.468
Plateau airway pressure; cmH_2_O	24 (22–27 [12–41]	24 (22–27 [12–41])	27 (27–29 [26–31])	0.048
Missing	54	52	2	
Peak airway pressure; cmH_2_O	25 (20–30 [2–41])	25 (20–29 [2–41])	29 (27–31 [11–31])	0.171
Missing	54	52	2	
Tidal volume based on predicted body weight; ml.kg^‐1^	7.2 (2.1)	7.2 (2.0)	7.1 (3.3)	0.978
Missing	4	2	0	
PaO_2_/F_I_O_2_	17 (13.6–21.3 [5.6–33.3])	17.5 (13.7–21.5 [5.6–33.3])	15.6 (12.5–17.5 [10.1–24.4])	0.361
PEEP; cmH_2_O	9.8 (3.6)	9.9 (3.6)	8.7 (3.0)	0.351
Missing	1	1	0	
Respiratory rate; breaths.min^‐1^	24.3 (5.3)	24.2 (5.2)	25.3 (6.3)	0.676
Driving pressure; cmH_2_O	14.7 (7.1)	14.2 (7.1)	19.4 (5.1)	0.086
Missing	54	52	2	
Dynamic compliance; ml.cmH_2_O^‐1^	30 (20–40 [8–180])	32 (21–41 [8–180])	19 (17–20 [13–32])	0.031
Missing	54	52	2	
Murray lung injury score	3 [2–3 [1–4])	3 (2–3 [1–4])	2.9 (3–3 [2–4])	0.491
Missing	12	11	1	

RRT, renal replacement therapy.

**Table 4:** Clinical outcomes of patients with COVID‐19 whose lungs were mechanically ventilated at 30 days following echocardiography. Values are number (proportion).

	All n = 112	No or less than severe right ventricular dysfunction n = 105	Severe right ventricular dilation and septal flattening n = 7	p value
Death	53 (47%)	47 (45%)	6 (86%)	0.051
Renal replacement therapy	28 (25%)	25 (24%)	3 (43%)	0.581
Prone ventilation	55 (49%)	53 (51%)	2 (29%)	0.322
Referral for veno‐venous extracorporeal membrane oxygenation	15 (13%)	15 (14%)	0	0.663

**Table 5:** Firth's bias‐reduced logistic regression for 30‐day mortality in patients with COVID‐19 whose lungs were mechanically ventilated.

	Adjusted OR (95%CI)	p value
Severe right ventricular dilation and septal flattening	5.12 (0.99–51.38)	0.051
Age; y	1.48 (1.19–1.91)	0.001
Female sex	1.12 (0.46–2.73)	0.803
Non‐white ethnic origin	0.92 (0.22–3.54)	0.899
APACHE‐2 score on admission to ICU (per 5‐score increase)	1.27 (0.87–1.90)	0.221
Time from tracheal intubation to date of echocardiogram; days	1.01 (0.88–1.17)	0.837

Radiologically confirmed or clinically suspected pulmonary thromboembolic disease was present in 57% of patients with right ventricular dysfunction compared with 5% in those without (p < 0.001). In keeping with this, treatment with anticoagulation was more common in those with right ventricular dysfunction (Table 3). There was no difference in: platelet count; prothrombin time; activated partial thromboplastin time; and D‐dimer levels between the two groups (Table 3). Where it could be measured, plateau airway pressure was higher and pulmonary compliance was lower in patients with right ventricular dysfunction (Table 3). There was no difference in: peak airway pressure; positive end expiratory pressure; driving pressure; or indexed tidal volume between the groups (Table 3). There was no difference in PaO_2_/F_I_O_2_ ratio, requirement for prone positioning in the previous 24 h or Murray lung injury score [15] between the groups (Table 3).

**Figure 3:**
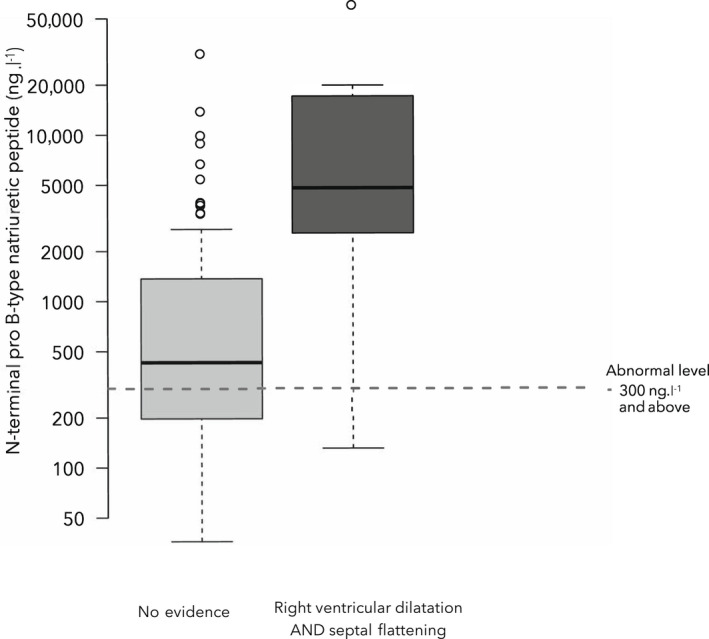
N‐terminal pro B‐type natriuretic peptide levels in of patients with COVID‐19 whose lungs were mechanically ventilated with and without right ventricular dysfunction.

There was no difference in the proportion of patients with normal or abnormal NT‐proBNP levels, between those with and without right ventricular dysfunction (Table 3). However, median NT‐proBNP values were higher in those patients with right ventricular dysfunction (Fig. 3). Conversely, there was no difference in troponin levels (I or T) between the groups (Table 3). However, raised troponin values were more frequent in patients with right ventricular dysfunction. Given the low frequency of the combination of severe right ventricular dilation and septal flattening (the primary outcome), on an exploratory basis, an alternative outcome describing the presence of severe right ventricular dilation and/or interventricular flattening was examined. This occurred in 33 (30%) patients (Fig. 2). Subjective right ventricular dysfunction was present in 44% of patients with this definition of right ventricular dysfunction, compared with 3% without (p < 0.001). Like the primary outcome definition of right ventricular dysfunction, patients with the alternative outcome were more likely to have confirmed or suspected thromboembolic disease, lower base excess and higher plateau airway pressure (see online Supporting Information Tables S2–S4). However, in contrast to the primary outcome, patients with the alternative outcome had: a higher sequential organ failure assessment score on the day of echocardiography; higher activated partial thromboplastin time; increased requirement for vasopressors; and lower PaO_2_/F_I_O_2_ ratio (see online Supporting Information Table S4). There was also no association with the phase of disease regarding time from symptom onset to tracheal intubation (see online Supporting Information Table S2). Mortality in this group was 52%, in contrast to 46% in those with no evidence of right ventricular dysfunction (p = 0.679, online Supporting Information Table S5). Regression analyses controlling for baseline characteristics, phase of disease and baseline severity of illness demonstrated the presence of this definition of right ventricular dysfunction was not independently associated with 30‐day mortality (OR 1.35 (95%CI 0.54–3.46); p = 0.523) (see online Supporting Information Table S6).

## Discussion

This is one of the first studies to prospectively explore the prevalence of right ventricular dysfunction in patients with COVID‐19 whose lungs are mechanically ventilated and its association with mortality. The prevalence of right ventricular dysfunction was 6% and this was associated with a mortality of 86%, in contrast to 45% in those without right ventricular dysfunction. A universal definition of right ventricular dysfunction has not yet been well established. It has been suggested the term refers to structural changes (abnormal imaging and/or biomarkers) but with maintained cardiac output, a clinical setting which may progress to fulminant right ventricular failure. Right ventricular failure is characterised by insufficient delivery of blood from the right ventricle along with elevated systemic venous pressures [16]. As right ventricular failure is a challenging clinical diagnosis to make, we referred to echocardiographic changes as right ventricular dysfunction. There is, however, a high likelihood that included patients with a diagnosis of right ventricular dysfunction would also have fitted the clinical criteria for right ventricular failure, where there was associated tachycardia, evidence of systemic hypoperfusion (disordered acid–base status), renal dysfunction and increased mortality.

Echocardiographic assessment of right ventricular function is challenging due to its complex geometry, retrosternal position and marked load dependence. As a result, there is no gold standard quantitative measurement of right ventricular function. International guidelines advocate assessment should incorporate a combination of both qualitative and quantitative parameters [17]. These quantitative methods, however, have been observed to vary markedly in their diagnostic performance [18]. More importantly, for our study, they are not assessed as part of a focused, bed‐side FICE scan. We deliberately selected a pragmatic definition of right ventricular dysfunction to empower bed‐side critical care clinicians to make the diagnosis [19]. Identification of right ventricular dysfunction could inform patient prognosis, alert to the potential for associated pathologies, such as thromboembolic disease, and influence treatment strategies such as optimisation of mechanical ventilation and introduction of inotropic support or pulmonary vasodilators. Furthermore, should any interventional study of right ventricular therapies be proposed in this patient group, it is imperative that any inclusion criteria allow the treating bed‐side clinician to make the diagnosis.

The echocardiographic combination of right ventricular dilation and septal flattening is commonly referred to as acute cor pulmonale and is thought to demonstrate a severe form of right ventricular dysfunction with evidence of both pressure and volume overload [20]. The observed prevalence of right ventricular dysfunction seen in the current study at 6% was lower than hypothesised. In the early phases of the pandemic, anecdotal clinical reports and early peer‐reviewed literature suggested right ventricular dysfunction was very common [21]. These early studies had mechanical ventilation rates of only 10–30% and it was hypothesised the prevalence of right ventricular dysfunction would be higher in a cohort requiring tracheal intubation and mechanical ventilation. Previous reports of right ventricular dysfunction in ARDS described a prevalence of up to 50% [10, 22].

The lower‐than‐expected prevalence of right ventricular dysfunction may have been observed for several reasons. First, these early, observational and retrospective reports are likely to have been influenced by both ascertainment and selection bias, where assessments were only performed for clinical purposes, in all likelihood in patients who were haemodynamically unstable or deteriorating, meaning prevalence was over‐reported [21]. We recruited 24% of all patients admitted to participating ICUs during the study period; the prospective and systematic approach to patient recruitment provides confidence that the prevalence presented here is accurate. When compared with contemporaneous national reporting by both the Intensive Care National Audit and Research Centre and the Scottish Intensive Care Society Audit Group, patients recruited in our study are representative in terms of severity of illness and mortality [23, 24]. Second, there were significant differences in baseline characteristics, clinical care and outcomes between the time of these early reports and the UK second wave when our cohort was recruited. Attitudes to non‐invasive respiratory support, ventilation practice, fluid management and anticoagulation all evolved and successful drug therapies were discovered and implemented. For example, 66% of participants were treated with intravenous steroids before tracheal intubation [25, 26]. Third, the definition of right ventricular dysfunction used in this study, necessitating the presence of both severe dilation and septal flattening, is a more stringent definition than that used in other reports. The alternative outcome we employed was found in 30% of patients, while subjective right ventricular dysfunction was diagnosed in 14%. This is consistent with previous studies in COVID‐19, where right ventricular dilation was reported to be present in 31–52% of cases [2, 4, 5, 11].

Our findings are in keeping with previous reports in ARDS. In a study of 752 patients by Mekontso Dessap et al. [10], acute cor pulmonale was present in 22% (95%CI 19–25%) and severe acute cor pulmonale (the same definition as used in our study) was present in 7% (95%CI 5–9%). As in our study, hospital mortality was only higher in patients with severe acute cor pulmonale when compared with all other patients (57% vs. 42%; p = 0.03), suggesting this definition of right ventricular dysfunction identifies a population of patients where the diagnosis yields significant clinical sequelae. High driving pressure, low PaO_2_/F_I_O_2_ ratio and high PaCO_2_ have been associated with right ventricular dysfunction in patients with ARDS [10]. Although we were not able reproduce these findings, our study demonstrates that plateau airway pressure was higher and compliance lower in those with right ventricular dysfunction, suggesting interplay between ARDS and the conduct of mechanical ventilation in the aetiology of right ventricular dysfunction in this population.

The observed association between confirmed or suspected thromboembolic disease and right ventricular dysfunction is not unexpected and demonstrates the role of macrothrombi in right ventricular dysfunction. It has been consistently demonstrated that patients with COVID‐19 in ICU have a high frequency of thrombotic complications with an incidence of 13% [27]. Right ventricular dysfunction is common in patients with thromboembolic disease and is a major determinant of short‐term survival [28]. Cardiomyocyte injury, quantified by elevated troponin levels, and haemodynamic cardiac stress, as quantified by increased natriuretic peptide concentrations, have been described both in COVID‐19 and ARDS [29, 30]. The association of elevated plasma NT‐proBNP and abnormal troponin levels with right ventricular dysfunction in this cohort suggests a potential role of direct myocardial injury in the aetiology of myocardial dysfunction in patients with COVID‐19.

Our study used a single time‐point of echocardiographic assessment and it is unknown whether patients classified as not having right ventricular dysfunction may have had abnormal echocardiography if imaging had occurred at a different time‐point during their ICU admission. Although we have precisely estimated the prevalence of right ventricular dysfunction in patients with COVID‐19 whose lungs are mechanically ventilated, the low number of patients fulfilling the primary outcome prevents detailed multivariate assessment of the factors associated with right ventricular dysfunction. In addition, the associations explored between right ventricular dysfunction and aetiological factors are at risk of type 1 and 2 errors, meaning they can only be considered exploratory in nature.

In conclusion, we have demonstrated that the prevalence of right ventricular dysfunction is lower than predicted in patients with COVID‐19 whose lungs are mechanically ventilated but that there is an association between this and mortality. We observed an association between right ventricular dysfunction and each of the aetiological domains of: ARDS; mechanical ventilation; thromboembolic disease; and myocardial injury. This serves to highlight the need for increased clinician awareness of right ventricular dysfunction and aids the design of therapeutic studies seeking to improve outcomes in this critically ill patient group.

## Supporting information


**Table S1.** Differences in mortality rates.
**Table S2**. Patient characteristics at ICU admission for the post‐hoc outcome.
**Table S3**. Measured echocardiographic variables for the post‐hoc outcome.
**Table S4**. Patient characteristics on the day of echocardiography for the post‐hoc outcome.
**Table S5**. Clinical outcomes of included patients at 30 days following echocardiography for the post‐hoc outcome.
**Table S6**. Firth’s bias reduced logistic regression for 30‐day mortality for the post‐hoc outcome.

## Appendix 1 COVID‐RV co‐investigators.

A Reece, C Kitchen, M Gillies, V Dabek, V Irvine, COVID‐RV trial management group, Glasgow, UK.

J MacBrayne, K Sim, T Scott, E Trumper, F Savage, A Allan, J Falconer, A Coutts, Aberdeen Royal Infirmary, Aberdeen, UK.

A McDonald, J Rutherford, D Christie, C Jardine, Dumfries & Galloway Royal Infirmary, Dumfries, UK.

M Hughes, S Cathcart, Glasgow Royal Infirmary, Glasgow, UK.

M Sim, B Docking, M Thornton, Queen Elizabeth University Hospital, Glasgow, UK.

B Greatorex, J Rae, C Barr, C Bradley, F Barrett, R Campbell, N Clarke, M Mascarenhas, J Matheson, D McDonald, M O'Hara, L O'keeffe Raigmore Hospital, Inverness, UK.

R Price, M McHendry, Royal Alexandra Hospital, Paisley, UK.

D McLaughlan, C Herman, H Elliot, S Meehan, University Hospital Ayr, Ayr, UK.

D Finn, G Brannan, S Wood, T Watson, K Ross, University Hospital Crosshouse, Kilmarnock, UK.

N Tatarkowska, R Boyle, E Lee, University Hospital Hairmyres, East Kilbride, UK.

A Morrison, P. Lucie, C Lochrin, S Clements, D Vigni, University Hospital Wishaw, Wishaw, UK.
