# Supplementary material for: Right ventricular dysfunction in patients with COVID‐19 pneumonitis whose lungs are mechanically ventilated: a multicentre prospective cohort study
Source: Anaesthesia. 2022 May 24;77(7):772–84. doi: 10.1111/anae.15745 (PMC9322018; doi:10.1111/anae.15745)
Supplement: Supplementary file 1 — Table S1. Differences in mortality rates. Table S2. Patient characteristics at ICU admission for the post‐hoc outcome. Table S3. Measured echocardiographic variables for the post‐hoc outcome. Table S4. Patient characteristics on the day of echocardiography for the post‐hoc outcome. Table S5. Clinical outcomes of included patients at 30 days following echocardiography for the post‐hoc outcome. Table S6. Firth’s bias reduced logistic regression for 30‐day mortality for the post‐hoc outcome. [file ANAE-77-772-s001.docx]

| **Supplementary Table 1. Indicative power. Differences in mortality rates that could be detected at a 5% significance level for sample sizes of 120 and 150, and proportions of patients with RV dysfunction of 25% and 50%, assuming an overall mortality rate of 50%.** | | | | | | |
| --- | --- | --- | --- | --- | --- | --- |
| **Sample Size** | **Power** | **Number *without* RV dysfunction** | **Number *with* RV dysfunction** | **Mortality in patients *without* RV dysfunction** | **Mortality in patients *with* RV dysfunction** | **Odds Ratio** |
| 120 | 80% | 60 | 60 | 0.373 | 0.627 | 2.83 |
| 120 | 90% | 60 | 60 | 0.354 | 0.646 | 3.33 |
| 150 | 80% | 75 | 75 | 0.386 | 0.614 | 2.53 |
| 150 | 90% | 75 | 75 | 0.369 | 0.631 | 2.92 |
| 120 | 80% | 90 | 30 | 0.427 | 0.719 | 3.43 |
| 120 | 90% | 90 | 30 | 0.417 | 0.749 | 4.17 |
| 150 | 80% | 112 | 38 | 0.435 | 0.695 | 2.96 |
| 150 | 90% | 112 | 38 | 0.426 | 0.722 | 3.50 |
| RV, Right Ventricle. | | | | | | |

| **Supplementary Table 2. Patient characteristics at ICU admission. Values are mean (SD), number (proportion) or median (IQR [range]).** | | | | | | | | |
| --- | --- | --- | --- | --- | --- | --- | --- | --- |
|  | | | |  |  | **ALTERNATIVE ENDPOINT** | |  |
|  | | | | **All Patients** |  | **No RV dysfunction** | **RV dysfunction**  **(Severe RV dilatation *and/or* septal flattening)** |  |
|  | | | | **(n=112)** |  | **(n=79)** | **(n=33)** | ***p*** |
|  | **Age, years** | | | 59.2 (11.3) |  | 58.5 (11.8) | 60.7 (9.9) | 0.326 |
|  | **Sex, Male** | | | 74 (66%) |  | 52 (66%) | 22 (67%) | >0.99 |
|  | **BMI, kg m^-2^** | | ***n (n missing)*** | *110 (2)* |  | 78 (1) | 32 (1) |  |
|  | | |  | 32.9 (7.1) |  | 32.8 (7.3) | 32.9 (6.7) | 0.952 |
|  | **Ethnicity** | **White** | | 100 (89%) |  | 74 (94%) | 26 (79%) |  |
|  | | **Non-white** | | 12 (11%) |  | 5 (6%) | 7 (21%) | 0.039 |
|  | **Time since symptom onset to intubation, days** | | | 11 (7-16 [0-46]) |  | 10.5 (7.2-14 [0-46]) | 11 (7-17 [3-33]) | 0.384 |
|  | **Clinical frailty score** | | ***n (n missing)*** | *111 (1)* |  | 78 (1) | 33 (0) |  |
|  | | |  | 2 (2-3 [1-5]) |  | 2 (2-3 [1-5]) | 2 (2-3 [1-5]) | 0.930 |
|  | **APACHE II** | | ***n (n missing)*** | *107 (5)* |  | 74 (5) | 33 (0) |  |
|  | | |  | 16.6 (5.8) |  | 16.6 (6.2) | 16.8 (5.1) | 0.877 |
|  | **CCCC** | | ***n (n missing)*** | *102 (10)* |  | 73 (6) | 29 (4) |  |
|  | | |  | 10.2 (2.7) |  | 10.0 (3) | 10.8 (1.9) | 0.105 |
| ***Comorbidities*** | | | | | | | | |
|  | **Smoking** | **Non-smoker** | | 63 (56%) |  | 41 (52%) | 22 (67%) | 0.293 |
|  | | **Ex-smoker > 1 year** | | 40 (36%) |  | 30 (38%) | 10 (30%) |  |
|  | | **Current or within 1 year** | | 9 (8%) |  | 8 (10%) | 1 (3%) |  |
|  | **Alcohol history** | ***n (n missing)*** | | *110 (2)* |  | 77 (2) | 33 (0) | 0.544 |
|  |  | **None** | | 34 (31%) |  | 24 (31%) | 10 (30%) |  |
|  | | **Minimal** | | 57 (52%) |  | 37 (48%) | 20 (61%) |  |
|  | | **Moderate** | | 8 (7%) |  | 7 (9%) | 1 (3%) |  |
|  | | **Excess** | | 11 (10%) |  | 9 (12%) | 2 (6%) |  |
|  | **Hypertension** | | | 38 (34%) |  | 25 (32%) | 13 (39%) | 0.513 |
|  | **Coronary artery disease** | | | 11 (10%) |  | 7 (9%) | 4 (12%) | 0.333 |
|  | **Diabetes** | | | 33 (30%) |  | 24 (30%) | 9 (27%) | 0.823 |
|  | **Asthma** | | | 16 (14%) |  | 12 (15%) | 4 (12%) | 0.775 |
|  | **COPD** | | | 10 (9%) |  | 8 (10%) | 2 (6%) | 0.803 |
| ***Treatments before intubation*** | | | | | | | | |
|  | **Intravenous corticosteroids** | | | 74 (66%) |  | 49 (62%) | 25 (76%) | 0.193 |
|  | **Non-invasive ventilation** | | | 76 (68%) |  | 54 (68%) | 22 (67%) | >0.99 |
|  | **High flow nasal oxygen** | | | 65 (58%) |  | 41 (52%) | 24 (73%) | 0.058 |
|  | **Awake self-proning** | | | 57 (51%) |  | 38 (48%) | 19 (58%) | 0.411 |
| ***Acute comorbidities since hospital admission*** | | | | | | | | |
|  | **New arrhythmias** | | | 17 (15%) |  | 11 (14%) | 6 (18%) | 0.573 |
|  | **Confirmed or suspected PTE** | **Radiologically confirmed** | | 4 (4%) |  | 0 (0%) | 4 (12%) | 0.010 |
|  |  | **Clinically suspected** | | 5 (5%) |  | 3 (4%) | 2 (6%) |  |
|  |  | **No** | | 101 (90%) |  | 74 (94%) | 27 (82%) |  |
|  |  | **Unknown** | | 2 (2%) |  | 2 (3%) | 0 (0%) |  |
|  | **ACS** | | | 5 (5%) |  | 3 (4%) | 2 (6%) | 0.630 |
|  | **Requirement for RRT** | | | 18 (16%) |  | 12 (15%) | 6 (18%) | 0.779 |
| RV, Right Ventricle; BMI, Body Mass Index; APACHE, Acute Physiology And Chronic Health Evaluation; CCCC, Coronavirus Clinical Characterisation Consortium; COPD, Chronic Obstructive Pulmonary Disease; PTE, Pulmonary Thromboembolism; ACS, Acute Coronary Syndrome; RRT, Renal Replacement Therapy. | | | | | | | | |

| **Supplementary Table 3. Echocardiography.**  **Data are presented as median (IQR [range]) or n (%). Data are complete unless indicated by n (n missing).** | | | | | | |
| --- | --- | --- | --- | --- | --- | --- |
|  | |  |  | **ALTERNATIVE ENDPOINT** | |  |
|  | | **All** |  | **No RV dysfunction** | **RV dysfunction**  **(Severe RV dilatation *and/or* septal flattening)** |  |
|  | | **(n=112)** |  | **(n=79)** | **(n=33)** | ***p*** |
| **Time from symptom onset to echocardiography, days** | ***n (n missing)*** |  |  | 78 (1) | 33 (0) |  |
|  |  | 18 (13-22 [3-51]) |  | 17.5 (13-21 [3-51]) | 18 (13-24 [9-37] | 0.288 |
| **Time from intubation to echocardiography, days** | | 5 (4-8 [2-14]) |  | 5 (3.2-8 [2-14]) | 5 (4-7 [2-12]) | 0.777 |
| **RV dilatation** | ***n (n missing)*** | 110 (2) |  | 77 (2) | 33 (0) |  |
|  |  | 31 (28%) |  | 0 (0%) | 31 (94%) | - |
| **Septal flattening** | ***n (n missing)*** | 109 (3) |  | 76 (3) | 33 (0) |  |
|  |  | 9 (8%) |  | 0 (0%) | 9 (27%) | - |
| **Subjective LV dysfunction** | ***n (n missing)*** | 110 (2) |  | 79 (0) | 31 (2) |  |
|  |  | 12 (11%) |  | 6 (8%) | 6 (19%) | 0.093 |
| **Subjective RV dysfunction** | ***n (n missing)*** | 111 (1) |  | 79 (0) | 32 (1) |  |
|  |  | 16 (14%) |  | 2 (3%) | 14 (44%) | <0.001 |
| RV, Right Ventricle; LV, Left Ventricle. | | | | | | |

| **Supplementary Table 4. Patient characteristics on day of echocardiography.**  **Data are presented as mean (SD), median (IQR [range]) or n (%). Data are complete unless indicated by n (n missing).** | | | | | | | | |
| --- | --- | --- | --- | --- | --- | --- | --- | --- |
|  | | | |  |  | **ALTERNATIVE ENDPOINT** | |  |
|  | | | | **All** |  | **No RV dysfunction** | **RV dysfunction**  **(Severe RV dilatation *and/or* septal flattening)** |  |
|  | | | | **(n=112)** |  | **(n=79)** | **(n=33)** | ***p*** |
|  | **Requirement for prone invasive ventilation since ICU admission** | | | 79 (71%) |  | 51 (65%) | 28 (85%) | 0.061 |
|  | **SOFA score** | | | 7.9 (3) |  | 7.6 (2.8) | 8.8 (3.2) | 0.048 |
|  | **Requirement for RRT on day of echo** | | | 15 (13%) |  | 8 (10%) | 7 (21%) | 0.135 |
| ***Lab Measurements*** | | | | | | | | |
| ***Arterial Blood Gas*** | | | | | | | | |
|  | **[H^+^], nmol L^-1^** | | ***n (n missing)*** | *97 (15)* |  | 68 (11) | 29 (4) |  |
|  |  | |  | 39 (35.8-46 [27.7-68]) |  | 39 (34.8-45 [27.7-55]) | 40.9 (36-49 [31-68]) | 0.230 |
|  | **PaO_2_, kPa** | | | 9.3 (1.2) |  | 9.4 (1.2) | 9.1 (1.3) | 0.428 |
|  | **PaCO_2_, kPa** | | ***n (n missing)*** | *109 (3)* |  | 76 (3) | 33 (0) |  |
|  |  | |  | 6.9 (5.9-8 [4.1-13.1]) |  | 6.8 (5.9- 8 [4.1-11.3]) | 7 (5.9-7.9 [4.4-13.1]) | 0.929 |
|  | **BE, mmol** | | ***n (n missing)*** | *110 (2)* |  | 77 (2) | 33 (0) |  |
|  |  | |  | 5.9 (6.5) |  | 6.8 (6.3) | 3.8 (6.4) | 0.026 |
|  | **Bicarbonate, mmol L^-1^** | | ***n (n missing)*** | *109 (3)* |  | 76 (3) | 33 (0) |  |
|  |  | |  | 31.8 (6.6) |  | 32.5 (6.7) | 30.0 (6.1) | 0.063 |
| ***Full Blood Count*** | | | | | | | | |
|  | **Haemoglobin, g dl^-1^** | |  | 11 (1.8) |  | 11.2 (1.9) | 10.7 (1.4) | 0.127 |
|  | **Neutrophils, x10^9^ L^-1^** | |  | 10.5 (8.5-14.9 [2.5-43]) |  | 10.3 (8.4-13.6 [4.3-43]) | 12.3 (8.9-17.7 [2.5-28.3]) | 0.128 |
|  | **Lymphocytes, x10^9^ L^-1^** | |  | 0.9 (0.5-1.4 [0.1-4.5]) |  | 0.9 (0.4-1.3 [0.2-4.5]) | 0.9 (0.7-1.5 [0.1-2.4]) | 0.239 |
|  | **Platelets, x10^9^ L^-1^** | |  | 279.5 (109.5) |  | 280.8 (111.7) | 276.5 (105.7) | 0.849 |
| ***Inflammation*** | | | | | | | | |
|  | **CRP, mg L^-1^** | |  | 61.5 [11.8-157.5 [1-665]) |  | 54 (11-149 [1-446]) | 72 (14-178 [1-665]) | 0.375 |
| ***Coagulation*** | | | | | | | | |
|  | **D-Dimers, mg L^-1^ FEU** | | ***n (n missing)*** | *81 (31)* |  | 54 (25) | 27 (6) |  |
|  |  | |  | 1264 (601-2605 [1.1-30667]) |  | 1373 (724.8-2496 [163-30667]) | 1156 (504-2822 [1.1-12890]) | 0.745 |
|  | **PT, seconds** | |  | 12 (11-13.2 [9.8-26]) |  | 12 (11-13.2 [9.8-26]) | 13 (11-13 [10-17]) | 0.264 |
|  | **APTT, seconds** | | ***n (n missing)*** | *111 (1)* |  | 78 (1) | 33 (0) |  |
|  |  | |  | 27 (25-31 [18.6-263]) |  | 26 (24-30 [18.6-263]) | 29 (26-31 [22-69.3]) | 0.031 |
| ***Electrolytes*** | | | | | | | | |
|  | **Creatinine, μmol L^-1^** | |  | 69.5 (53.5-107 [27-396]) |  | 64 (53-99 [27-256]) | 84 (54-118 [34-396]) | 0.099 |
| ***Cardiac biomarkers*** | | | | | | | | |
|  | **NT-proBNP, ng L^-1^** | | ***n (n missing)*** | *100 (12)]* |  | 68 (11) | 32 (1) |  |
|  |  | |  | 458 (198.5-1689.5 [36-61280]) |  | 430.5 (194-1275.2 [36-10015]) | 537.5 (208-3634.2 [65-61280]) | 0.213 |
|  | ***Abnormal* NT-proBNP^A^** | | ***n (n missing)*** | *100 (12)* |  | 68 (11) | 32 (1) |  |
|  |  | |  | 63 (63%) |  | 43 (63%) | 20 (63%) | >0.99 |
|  | **hsTn I, ng L^-1^** | | ***n (n missing)*** | *64 (48)* |  | 40 (39) | 24 (9) |  |
|  |  | |  | 12 (4.8-42 [0-3585]) |  | 10 (4.8-40 [0-2044]) | 19.5 (4.8-42 [4-3585]) | 0.605 |
|  | **hsTn T, ng L^-1^** | | ***n (n missing)*** | *46 (66)* |  | 37 (42) | 9 (24) |  |
|  |  | |  | 16.5 [10-28.5 [0-473]) |  | 16 (10-26 [0-473]) | 18 (15-37 [10-92]) | 0.239 |
|  | ***Abnormal* troponin^B^** | | ***n (n missing)*** | *110 (2)* |  | 77 (2) | 33 (0) |  |
|  |  | |  | 51 (46%) |  | 34 (44%) | 17 (52%) | 0.535 |
| ***Clinical Parameters*** | | | | | | | | |
|  | **HR, bpm** | |  | 79 (19.9) |  | 78.1 (19.5) | 81.3 (21.1) | 0.446 |
|  | **Rhythm** | | **Sinus** | 107 (95.5%) |  | 76 (96.2%) | 31 (93.9%) |  |
|  |  | | **AF/Flutter** | 5 (5%) |  | 3 (4%) | 2 (6%) | 0.630 |
|  | **Mean BP, mmHg** | | ***n (n missing)*** | *109 (3)* |  | 77 (2) | 32 (1) |  |
|  |  | |  | 80.9 (13.4) |  | 82.1 (14.3) | 77.9 (10.5) | 0.089 |
|  | **CVP, cmH_2_O** | | ***n (n missing)*** | *74 (38)* |  | 52 (27) | 22 (11) |  |
|  |  | |  | 7 (4-12 [0-25]) |  | 7 (3.8-11.2 [0-20.4]) | 7.5 (6-13.8 [2-25]) | 0.307 |
| ***Drug Administration*** | | | | | | | | |
|  | **Vasopressors** | |  | 40 (36%) |  | 22 (28%) | 18 (55%) | 0.010 |
|  | **Inotropes** | |  | 1 (1%) |  | 0 (0%) | 1 (3%) | 0.295 |
|  | **Anticoagulation** | | **Prophylactic** | 93 (83%) |  | 69 (87%) | 24 (73%) | 0.057 |
|  |  | | **Therapeutic** | 17 (15%) |  | 8 (10%) | 9 (27%) |  |
|  |  | | **None** | 2 (2%) |  | 0 (0%) | 0 (0%) |  |
|  | **Paralysis** | |  | 52 (46%) |  | 34 (43%) | 18 (55%) | 0.511 |
| ***Ventilation*** | | | | | | | | |
|  | **FiO_2_** | |  | 0.6 (0.4-0.7 [0.3-1.0]) |  | 0.6 (0.4-0.7 [0.3-1.0]) | 0.6 (0.5-0.7 [0.4-0.9]) | 0.083 |
|  | **Requirement for prone ventilation in previous 24 hours** |  | | 44 (39%) |  | 28 (35%) | 16 (49%) | 0.498 |
|  | **Plateau airway pressure, cmH_2_O** | | ***n (n missing)*** | 58 (54) |  | 39 (40) | 19 (14) |  |
|  |  | |  | 24 (22-27 [12-41]) |  | 24 (22-26 [12-41]) | 27 (25-29.5 [18-32]) | 0.018 |
|  | **PAP, cmH_2_O** | | ***n (n missing)*** | *58 (54)* |  | 76 (3) | 33 (0) |  |
|  |  | |  | 25 (20-30 [2-41]) |  | 24 (20-28.2 [2-41]) | 27 (21-30 [11-35]) | 0.176 |
|  | **Tidal volume, ml kg^-1^ (PBW)** | | ***n (n missing)*** | *108 (4)* |  | 76 (3) | 32 (1) |  |
|  |  | |  | 7.2 (2.1) |  | 7.2 (2) | 7.2 (2.5) | 0.964 |
|  | **P/F ratio** | |  | 17 (13.6-21.3 [5.6-33.3]) |  | 17.8 (14.3-21.4 [5.6-33.3]) | 15.2 (11.7-18 [8.9-29.2]) | 0.047 |
|  | **PEEP, cmH_2_O** | | ***n (n missing)*** | *111 (1)* |  | 78 (1) | 33 (0) |  |
|  |  | |  | 9.8 (3.6) |  | 9.7 (3.7) | 10.2 (3.4) | 0.447 |
|  | **Resp rate (minute^-1^)** | |  | 24.3 (5.3) |  | 24.1 (5.3) | 24.8 (5.2) | 0.489 |
|  | **Driving pressure, cmH_2_O** | | ***n (n missing)*** | *58 (54)* |  | 39 (40) | 19 (14) |  |
|  |  | |  | 14.7 (7.1) |  | 14.0 (7.6) | 15.9 (5.7) | 0.290 |
|  | **Dynamic compliance, ml cmH_2_O^-1^** | | ***n (n missing)*** | *58 (54)* |  | 39 (40) | 19 (14) |  |
|  |  | |  | 29.8 (20-40 [8.4-180]) |  | 34.3 (22.7-41.8 [10-180]) | 21.3 (17.9-36 [8.4-150]) | 0.082 |
|  | **Murray lung injury score** | | ***n (n missing)*** | 100 (12) |  | 69 (10) | 31 (2) |  |
|  |  | |  | 2.8 (2.3-3 [0.7-3.8]) |  | 2.8 (2.3-3 [0.7-3.8]) | 2.8 (2.5-3.3 [1.5-3.8]) | 0.491 |
| A, NT-proBNP ≥300 ng L^-1^  B, hsTnT ≥15 ng L^-1^ or hsTnI ≥34 ng L^-1^ for males; ≥16 ng L^-1^ for females  RV, Right Ventricle; RRT, Renal Replacement Therapy; BE, Base Excess; CRP, C-Reactive Protein; PT, Prothrombin Time; APTT, Activated Partial Thromboplastin Time; NT-proBNP, N-terminal pro B-type Natriuretic Peptide; hsTn, High Sensitivity Troponin; HR, Heart Rate; AF, Atrial Fibrillation; BP, Blood Pressure; CVP, Central Venous Pressure; FiO_2_, Fraction of Inspired Oxygen; PAP, Peak Airway Pressure; PBW, Predicted Body Weight; PEEP, Positive End Expiratory Pressure. | | | | | | | | |

| **Supplementary Table 5. Clinical Consequences.**  **Data are presented as n (%).** | | | | | | |
| --- | --- | --- | --- | --- | --- | --- |
|  | |  |  | **ALTERNATIVE ENDPOINT** | |  |
|  | | **All** |  | **No RV dysfunction** | **RV dysfunction**  **(Severe RV dilatation *and/or* septal flattening)** |  |
|  | | **(n=112)** |  | **(n=79)** | **(n=33)** | ***p*** |
| ***30-day follow-up*** | | | | | | |
|  | **Death** | 53 (47%) |  | 36 (46%) | 17 (52%) | 0.679 |
|  | **RRT** | 28 (25%) |  | 19 (24%) | 9 (27%) | 0.879 |
|  | **Prone ventilation** | 55 (49%) |  | 42 (53%) | 13 (39%) | 0.213 |
|  | **Referral for ECMO** | 15 (13%) |  | 14 (18%) | 1 (3%) | 0.097 |
| RV, Right Ventricle; RRT, Renal Replacement Therapy; ECMO, Extra Corporeal Membrane Oxygenation. | | | | | | |

| **Supplementary Table 6. Logistic regression model predicting 30-day mortality adjusting for remaining variables in table** | | |
| --- | --- | --- |
|  | **OR (95% CI)** | ***p*** |
| **RV dysfunction (dilatation *and/or* septal flattening)** | 1.35 (0.54, 3.46) | 0.523 |
| **Age, in years (per 5-year increase)** | 1.52 (1.21, 1.97) | 0.001 |
| **Female Gender** | 1.09 (0.44, 2.70) | 0.855 |
| **Non-white ethnicity** | 0.68 (0.15, 2.90) | 0.610 |
| **APACHE II score on admission to ICU (per 5-score increase)** | 1.30 (0.88, 1.98) | 0.191 |
| **Time from intubation to date of echo, in days (per 1-day increase)** | 1.02 (0.88, 1.17) | 0.826 |
| RV, Right Ventricle, APACHE, Acute Physiology And Chronic Health Evaluation | | |
